# Diabetic Retinopathy and NADPH Oxidase-2: A Sweet Slippery Road

**DOI:** 10.3390/antiox10050783

**Published:** 2021-05-15

**Authors:** Renu A. Kowluru

**Affiliations:** Department of Ophthalmology, Visual and Anatomical Sciences, Kresge Eye Institute, Wayne State University, Detroit, MI 48201, USA; rkowluru@med.wayne.edu; Tel.: +1-313-993-6714

**Keywords:** diabetic retinopathy, hyperlipidemia, mitochondria, NADPH oxidase

## Abstract

Diabetic retinopathy remains the leading cause of vision loss in working-age adults. The multi-factorial nature of the disease, along with the complex structure of the retina, have hindered in elucidating the exact molecular mechanism(s) of this blinding disease. Oxidative stress appears to play a significant role in its development and experimental models have shown that an increase in cytosolic Reacttive Oxygen Speies (ROS) due to the activation of NADPH oxidase 2 (Nox2), is an early event, which damages the mitochondria, accelerating loss of capillary cells. One of the integral proteins in the assembly of Nox2 holoenzyme, Rac1, is also activated in diabetes, and due to epigenetic modifications its gene transcripts are upregulated. Moreover, addition of hyperlipidemia in a hyperglycemic milieu (type 2 diabetes) further exacerbates Rac1-Nox2-ROS activation, and with time, this accelerates and worsens the mitochondrial damage, ultimately leading to the accelerated capillary cell loss and the development of diabetic retinopathy. Nox2, a multicomponent enzyme, is a good candidate to target for therapeutic interventions, and the inhibitors of Nox2 and Rac1 (and its regulators) are in experimental or clinical trials for other diseases; their possible use to prevent/halt retinopathy will be a welcoming sign for diabetic patients.

## 1. Introduction

Diabetes has become one of the fastest growing health challenges of the 21st century, and its global prevalence among adults has tripled in 20 years (151 million patients in 2000 to 463 million in 2019 (International Diabetes Federation, IDF Diabetes Atlas, 9th Edition). Although type 1 diabetes, where the body does not produce enough insulin, is the major cause of diabetes in childhood, around 90% patients worldwide have type 2 diabetes where insulin resistance is the major problem. Globally, due to a sedentary lifestyle and obesity, the prevalence of pre-diabetes (intermediate state of hyperglycemia with glucose levels above the normal range but below the diagnostic levels of diabetes) and type 2 diabetes is rising at an alarming rate [1,2]. 

Constant circulation of high glucose in diabetic patients affects the entire body resulting in many microvascular and macrovascular complications. One of the most feared microvascular complications of diabetes is retinopathy, which is the leading cause of blindness in working age-adults [3]. With the incidence of diabetes increasing at an alarming rate, the prevalence of blindness caused by diabetic retinopathy is also increasing worldwide. In 2005, an estimated 5.5 million people over the age of 40 had diabetic retinopathy, and this number is predicted to rise to 16 million by 2050 [4]. Diabetic retinopathy is a progressive disease, and sustained bathing of the highly vascularized retina, the light-sensitive layer of the eye which is responsible for converting light signal into neural signal to send it to the brain, damages its vasculature in high glucose leading it to swell and leak. The risk of damage increases with the duration of diabetes and the severity hyperglycemia, and new blood vessels begin to grow [3,5]. Therapeutic interventions for this sight-threatening disease, however, have been very limited. Despite some inherent shortcomings, vascular leakage is routinely stopped/slowed down by focal laser treatment, and neovascularization by pan-retinal coagulation [6]. Growth of new blood vessels is also now stopped/slowed down using anti-vascular endothelial growth factor (VEGF) therapies [7], but not all of the patients respond to these anti-VEGF treatments. In addition, due to the short half-life of anti-VEGF therapies (4–6 weeks), which requires frequent visits to the ophthalmologists, patient compliance remains one of the major issues.

Groundbreaking Diabetes Control and Complications Trials (DCCT) have demonstrated the benefits of reducing glycemic levels on the progression of diabetic complications including retinopathy, and even after three decades, control of glycemia remains one of the best options for a diabetic patient to prevent the progression of diabetic retinopathy [8]. However, many other systemic factors are also implicated in its development/progression including hypertension and hyperlipidemia. Lipid-lowering agents have shown benefits in preventing progression of diabetic retinopathy. The Action to Control Cardiovascular Risk in Diabetes (ACCORD) study has demonstrated reduced rate of progression of diabetic retinopathy with a combination of fenofibrate and simvastatin, and the Fenofibrate Intervention and Event Lowering in Diabetes (FIELD) study has shown a clear reduction in the frequency of laser photocoagulation by proliferative diabetic retinopathy [5,9,10,11]. Thus, the primary prevention strategy to prevent/ delay diabetic retinopathy, in addition to controlling blood sugar, also requires lifestyle modifications including dietary modifications and physical activities.

## 2. Pathogenesis of Diabetic Retinopathy

The pathogenesis of diabetic retinopathy is complex as the patient remains asymptomatic in the early stages of the disease. The earliest clinical signs, seen by an eye care provider, are microaneurysms and intraretinal hemorrhages, and as the disease progresses, the number and size of hemorrhages increase and precapillary arterioles occlude. Gradually, the capillaries become nonperfused, which results in neovascularization, and if left untreated, to retinal detachment and blindness [3]. Experimental models have provided better insights into its pathogenesis, and have shown that the basement membrane thickens, and apoptosis of the capillary cells, seen in the early stages of this progressive disease, results in degenerative capillaries and pericyte ghosts [3,12,13]. In addition to the vasculature, other cells of the retina including ganglion cells, also undergo accelerated apoptosis, and retinal functions (electroretinograms and contrast sensitivity) are impaired before any vascular changes can be seen [14].

Many leading laboratories have been trying to unveil the complex pathogenesis of this slow progressing blinding disease, but the molecular mechanism(s) still remains elusive. Several major metabolic abnormalities have been implicated in diabetic retinopathy; for example, accumulation of polyols due to conversion of excess circulating glucose to sorbitol by aldose reductase oxidizing NADPH to NAPP^+^ and activation of protein kinase C. Additionally, interactions of glucose with proteins or lipids, via nonenzymatic reactions, forming Schiff’s base and Amadori products, and they subsequently form advanced glycation end products. Circulating high glucose itself can auto-oxidize, accumulating reactive oxygen species (ROS) [15,16,17,18]. Polyunsaturated fatty acid rich retina is a desired target for oxidative damage [19], and in diabetes, ROS production is increased from both cytosolic and mitochondrial compartments of a cell, damaging the retina [20,21]. Oxidative stress, an imbalance between free radicals accumulation and their removal, is now considered as one of the major metabolic abnormalities associated with the development of diabetic retinopathy, and other major metabolic abnormalities including activation of polyol pathway and protein kinase C and advanced glycation end products, are also associated with increase in oxidative stress [16,22,23,24,25]. However, despite extensive research in the field, multifactorial nature of this progressive disease has made it difficult to identify a link between any specific abnormality and the development of diabetic retinopathy.

## 3. NADPH Oxidase

Historically ROS are viewed as by-products of oxidative metabolism in the mitochondria; however, they are also produced enzymatically by extra-mitochondrial sources including xanthine oxidase and NADPH oxidases (Nox). While xanthine oxidase generates free radicals by catalyzing the oxidation of xanthine and hypoxanthine during purine metabolism, Nox catalyze the production of free radicals by transferring one electron to oxygen from NADPH [26,27]. The Nox family has seven catalytic homologues, and although first identified as the enzymes responsible for respiratory burst, these enzymes are also expressed in a variety of nonphagocytic cells including endothelial cells and smooth muscle cells [28,29,30,31]. Nox-derived ROS affect many signaling processes and play essential roles in normal physiology and immune system [26]. Among this family of enzymes, Nox2 catalyzes one-electron reduction of oxygen to superoxide, and this multicomponent protein complex has both membrane and cytosolic components. It has two trans-membrane proteins (p22^phox^ and gp91^phox^/Nox2, which form the cytochrome b558) and three cytosolic proteins (p47^phox^, p67^phox^, p40^phox^). This spatial separation of the subunits allows it to remain dormant in resting cells, but in response to a stimulation, the cytosolic components migrate to the membrane where they assemble with the flavocytochrome b_558_ to form the holo-active enzyme. The cytosolic core also has a small G-protein, Ras-related C3 botulinum toxin substrate 1 (Rac1), which is integral in Nox2 activation [28]. p22^phox^ subunit requires protein–protein interactions, and p47^phos^ subunit moves from cytosol to the membrane, enabling recruitment of p67^phox^ and p40^phox^ subunits to the complex. Rac1 subsequently interacts with p67phox, and activation (GTP-bound) and deactivation (GDP-bound) cycle of Rac1 is mediated by specific guanine exchange factors (GEFs) and GTPase-activating proteins (GAPs), respectively. Under basal conditions, the guanine nucleotide-dissociation inhibitor (GDI) remains complexed with Rac1, preventing its constitutive activation and membrane targeting. Rac1 continues to cycle between the cytoplasm and the plasma membrane, and the hydrophilic cytoplasmic Rac1 lacks a transmembrane domain, but post-translational modifications (e.g., prenylation and lipidation) make it hydrophobic, allowing it to move to the plasma membrane. Post-translational modifications of Rac1 precisely dictate its functional regulation (GDI association) and subcellular localization [32,33,34,35,36,37].

In addition to being an integral component of the Nox2 holoenzyme, Rac1 also has nuclear functions including its cellular responses to genomic DNA damage and triggering activation of nuclear transcription factor [38,39], and nuclear sequestration of Rac1 is shown to affect its cytosolic functions [40,41]. Rac1 is also present in the mitochondria, where its response depends largely on the cell type. For example, in alveolar macrophages pulmonary fibrosis, mitochondrial import and direct electron transfer from cytochrome c to Rac1 modulates mitochondrial H_2_O_2_ production [42], and Bcl2 overexpression in lymphoma cells inhibits Rac1-mediated apoptosis [42]. Thus, Rac1 is not only restricted in the cytosol-membrane compartments, but it also has important functions in other subcellular organelles.

## 4. NADPH Oxidase and Diabetic Retinopathy

Nox2 is activated in the retina and its capillary cells in diabetes, and its activation, which is one of the major sources of cytosolic ROS, is an early event in this progressive disease [37,43]. Sustained production of cytosolic ROS, along with suboptimal antioxidant defense system including inhibition of cytosolic superoxide dismutase and glutathione peroxidase, allows continuous accumulation of ROS. This is further exacerbated by decreased levels of cytosolic antioxidant, glutathione [44,45]. The buildup of free radicals begins to damage the mitochondrial structural and functional stability, allowing cytochrome c to leak out in the cytosol to accelerate capillary cell apoptosis. Experimental models have demonstrated that in the pathogenesis of diabetic retinopathy, mitochondrial damage-capillary cell apoptosis precedes the development of histopathology characteristic of diabetic retinopathy [24,46]. Capillary cell loss also creates a hypoxic environment, which, by stimulating the burst of growth factors, results in neovascularization, and ultimately, in retinal detachment and blindness. Furthermore, ROS themselves also act as signaling molecules in growth factor-mediated physiological responses and play an important role in angiogenesis; enzymes of the Nox family are associated in the regulation of angiogenic signaling pathways of VEGF and hypoxia-inducible factors. Cell migration-proliferation-neovascularization is the hallmark of proliferative diabetic retinopathy [47], and Nox2 is shown to have a role in endothelial cell migration and proliferation [48,49]. Thus, Nox2 activation, which is an early event in the pathogenesis of diabetic retinopathy, contributes to both, background and proliferative stages of the disease.

### 4.1. Functional Activation of Nox2

As mentioned above, Nox2 holoenzyme has multi-components, and how diabetic milieu activates Nox2 is also equally complex. Activation of Rac1 is mediated by several factors, e.g., GEFs, GAPs and GDIs. GEFs facilitate GTP binding on Rac1 by releasing the bound GDP, and several GEFs are shown to govern Rac1 activation including T cell lymphoma invasion and metastasis (Tiam1) and Son of Sevenless 1 (Sos1). In diabetic retinopathy, Rac1 signaling is activated by Tiam1, which, with increase in duration of glucose insult, results in mitochondrial damage-capillary cell apoptosis; a specific inhibitor of Tiam1, NSC23766, inhibits glucose-induced mitochondrial damage and accelerated apoptosis of retinal capillary cells [31]. Inhibition of GEF Vav2, by its pharmacological inhibitor EHop, prevents activation of Rac1-Nox2-ROS signaling and inhibits mitochondrial damage and the development of retinopathy in diabetic mice [50]. Son of Sevenless homolog 1 (Sos1), another GEF, is also implicated in Rac1 activation in diabetic milieu; a regulator of Sos1, 66kDa proto-oncogene Src homologous-collagen homologue (p66Shc), regulates the of binding of Sos1 with the growth factor receptor-bound protein 2 (Grb2) [51], and in diabetes, the binding of Sos1 with Grb2 is decreased, resulting in Rac1 activation [52]. In contrast to GEFs, GDIs inhibit Rac1-GEF association to keep it in the cytosolic compartment [53], and in diabetic retinopathy the binding of Rac1 with GDI is decreased [50].

Function of Rac1 is also regulated by its post-translational modifications, and these modifications are implicated in its subcellular distribution and interaction with effector proteins. Prenylation (geranylgeranylation), palmitoylation and phosphorylation are some of the major modifications associated with Rac1 regulation. While Rac1 membrane translocation to form Nox2 holoenzyme is mediated by its prenylation, its association with GDIs is regulated by both palmitoylation and prenylation [54]. In diabetic retinopathy, both prenylation and palmitoylation are implicated in Rac1 activation, and inhibition of prenylation impedes glucose-induced Rac1-Vav2 association and Nox2 activation-mitochondrial damage, preventing capillary cell apoptosis. Localization of Rac1 in the lipid rafts is its major signaling site, and Rac1 translocation in the lipid rafts is mainly mediated by Rac1 palmitoylation. However, prenylation of Rac1 and an intact PBR (C-terminal polybasic region) region are essential for its palmitoylation [55]. In hyperglycemic milieu, inhibition of palmitoylation in retinal endothelial cells also prevents activation of Rac1-Nox2 signaling pathway [31,36,56].

Furthermore, Rac1 activation can also activate stress kinase p38MAPK, and activated stress kinase damages the mitochondria, resulting in the leakage of cytochrome c into the cytosol. This activates the apoptosis machinery, and accelerated apoptosis of retinal capillary cells leads to the degeneration of retinal capillaries and pericyte ghosts [50,57]. In addition, activated stress kinases can also breakdown the tight junctions and the blood-retinal barrier, the damages routinely seen during early stages of diabetic retinopathy [58,59,60,61]. Nox is also implicated in lipoxygenase-derived metabolites-mediated retinal endothelial cell migration and tube formation, the hallmarks of angiogenesis, seen in the proliferative stages of diabetic retinopathy [62]. Increased leukocyte adherence to the retinal microvasculature is commonly observed in animal models of diabetic retinopathy before they present histopathological lesions in their retinal microvasculature, and Nox-dependent activation of 12-HETE or 15-HETE is implicated in the leukocyte recruitment [63]. Thus, Nox2 activation in diabetes can results in the development of diabetic retinopathy via many different pathways including damaging the mitochondria by elevating ROS, breaking down blood-retina barrier and increasing leukostasis [64,65]. Nox enzymes also promote inflammation, inflammation plays a critical role in the development of diabetic retinopathy; increased inflammatory mediators including interleukin 1β, TNFα and intercellular adhesion molecule 1 are routinely seen in the retina in diabetes [64,66]. In addition, expression of Nox2 subunits is also influenced by angiotensin, and regulation of angiotensin-converting enzyme is shown to provide beneficial effect on the neurovascular pathology associated with diabetic retinopathy [67].

### 4.2. Transcriptional Activation of Rac1

Diabetes also upregulates the gene expression of *Rac1* [68,69], and recent research has shown that gene expression can also be regulated by epigenetic modifications, the modifications that regulate expression of a gene without altering its DNA sequence [70,71]. Epigenetic modifications These modifications can be transmitted to the daughter cells—thus epigenetic modifications can be considered as ‘inheritable’, which is not mediated via DNA sequence of genes [72]. However, depending on the regulation of external factors and life style, these epigenetic changes can also be erased/reversed, which makes them good therapeutic targets for chronic diseases [73]. These modifications are mainly influenced by external factors such as lifestyle, disease state and exposure to pollutants, and, as mentioned above, can be transmitted to the next generation, or can also be erased/reversed. Methylation of cytosine in the DNA, and modification of lysine or arginine in histones are some of the major epigenetic modifications. DNA methylation is associated with the closing of the chromatin structure, suppressing the gene expression, but the results of histone modifications depend on the type of the modification (e.g., methylation or acetylation), the site of methylation and the cell type [74,75,76]. Epigenetic modifications themselves are also interrelated; for example, histone methylation can direct DNA methylation patterns, and DNA methylation can influence histone modification patterns. Moreover, expression of the same gene can be regulated by single modification, or several modifications can function in concurrence [77,78].

**Figure 1:**
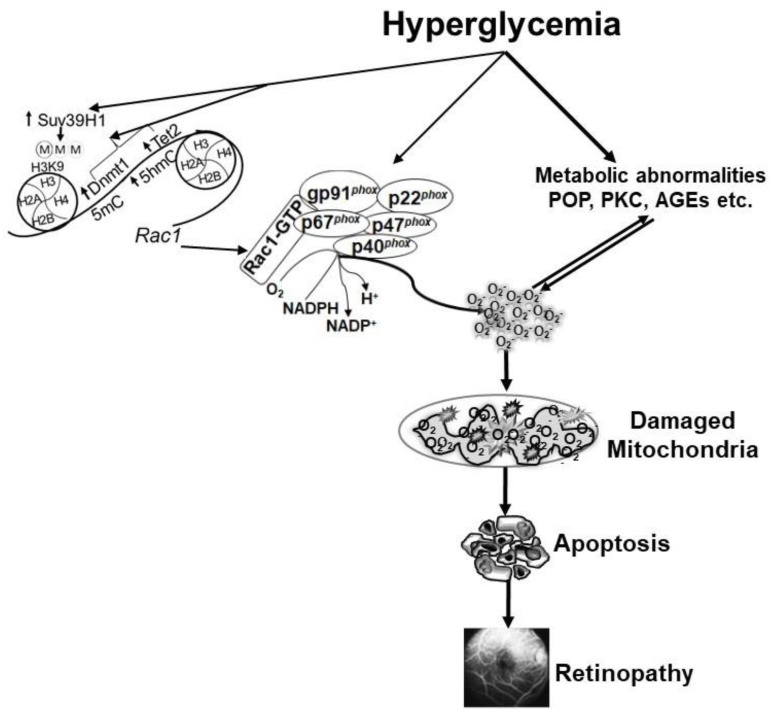
Hyperglycemia activates the key components of the Nox2 holoenzyme, and also increases *Rac1* transcription by modifying its DNA methylation and histone methylation. Activated Nox2 increases ROS production, and sustained increase in ROS damages the mitochondria. Capillary cell apoptosis is accelerated, which ultimately leads to the development of diabetic retinopathy. Hyperglycemia also induces many metabolic abnormalities that can generate ROS, and themselves are also influenced by ROS. POP = polyol pathway, PKC = protein kinase C and AGEs = advanced glycation end products.

DNA methylation is one of the most widely studied epigenetic modifications in various diseases, and formation of methyl cytosine (5mC) is considered as a mark of gene repression [79]. However, DNA methylation is a dynamic process, and the methylated cytosine can be rapidly hydroxylated, forming 5 methyl hydroxyl cytosine (5hmC), which is a mark of gene induction [80,81]. In diabetes, both DNA methylating (Dnmts) and hydroxymethylating/demethylating (ten-eleven translocase, Tets) enzymes are activated in the retinal vasculature [82], and *Rac1* promoter undergoes dynamic DNA methylation. While the binding of Dnmt1 isoform of the Dnmt family is increased, the levels of 5mC remain subnormal, but in contrast, 5hmC levels are upregulated at *Rac1* promoter, suggesting that despite activation of Dnmts, concomitant activation of Tets quickly converts 5mC to 5hmC. This interplay between two opposing enzymes leaves the promoter hypomethylated, which facilitates the binding of the transcription factor, resulting in transcriptional activation of *Rac1* [69]. In addition, DNA methylation can also influence histone methylation, and the recruitment of Dnmt1 at the promoter can be enabled by the methylation of lysine 9 of histone 3 (H3K9) [83]. In diabetes, the binding of histone trimethyltransferase, Suv39H1, is increased at *Rac1* promoter, and increase in H3K9me3 facilitates the recruitment of the DNA methylation machinery. Furthermore, regulation of Suv39H1, in addition to protecting H3K9 trimethylation, also protects active *Rac1* promoter DNA methylation-hydroxymethlation [84]. Thus, both DNA methylation and histone methylation function in concordance to regulate *Rac1* transcriptional activation (Figure 1).

## 5. Sweet and Slippery Road: NADPH Oxidase and Retinopathy in Type 1 and Type 2 Diabetic Patients

As mentioned above, about 90% of diabetic patients worldwide have type 2 diabetes. Although type 2 diabetes is more common in older adults, due to sedentary lifestyle and obesity in children it is becoming more prevalent in young adults. Nearly all patients with type 1 and 60% of patients with type 2 diabetes have retinopathy within 20 years of diabetes, but unfortunately, 20% of patients with type 2 diabetes have some form of retinopathy at the time of their diagnosis of diabetes, which may remain asymptomatic [85]. As mentioned earlier, hyperglycemia is considered as the main instigator of the development of diabetic retinopathy, but abnormalities in lipid metabolism are also now emerging as potential risk factors for its development, and lipid-lowering therapy has helped in reducing the number of laser treatments in patients with diabetes with proliferative retinopathy [5,9,10,11]. Results from a cohort of 1340 type 2 diabetic patients with prevalence of dyslipidemia in 83% of them have revealed a relationship between diabetic retinopathy and dyslipidemia [86]. In vitro and in vivo models of diabetic retinopathy have shown that simultaneous presence of both hyperlipidemia and hyperglycemia accelerates and exacerbates capillary cell apoptosis and the development of diabetic retinopathy; results from isolated retinal endothelial cells have documented that addition of lipotoxicity in a glucotoxic environment accelerates and exacerbates Nox2-ROS production, accelerating mitochondrial damage. An in vivo model of type 2 diabetes, Zucker Diabetic Fatty rat, has shown that Nox2-ROS mediated mtDNA damage and retinal vascular death are present as early as 20 weeks of age, when these rats are hyperglycemic for 14 weeks or less. In contrast, in type I model, mtDNA damage is not seen until the duration of diabetes is extended beyond 20 weeks [87,88]. Furthermore, a high fat-low streptozotocin type 2 diabetic model, which closely mimics type 2 diabetic populations, has shown that even during early stages of diabetes, compared to age-matched normal rats, Rac1-Nox2-ROS are high in type 1 diabetic animals, but type 2 diabetic animals have exacerbated increase in Rac1-Nox2-ROS, compared to type 1 diabetic model [43].

**Figure 2:**
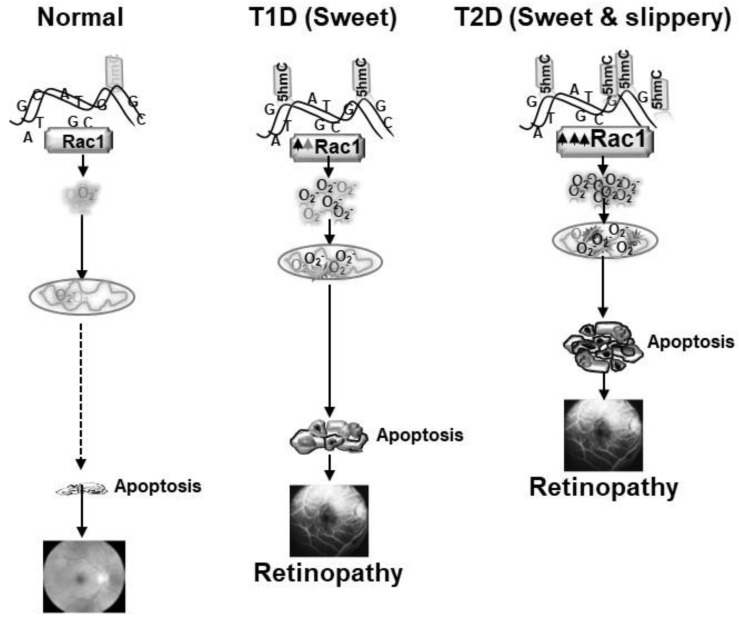
Exacerbated production of Rac1-Nox2- ROS in sweet and slippery (hyperglycemia and hyperlipidemia) milieu (type 2 diabetes, T2D) accelerates and exacerbates mitochondrial damage-capillary cell apoptosis, and the development of retinopathy, compared to sweet (hyperglycemic) milieu with mild to no hyperlipidemia (type 1 diabetes, T1D).

As mentioned above, epigenetic modifications at *Rac1* promoter are associated with its transcriptional activation in diabetes, and the promoter undergoes DNA methylation-hydroxymethylation. However, external factors including exercise and lifestyle, have major influence on epigenetic modifications, and lipids can also alter activities of the DNA methylation-hydroxymethylation enzymes. Our results from experimental models of type 1 and type 2 diabetes have shown that hyperglycemia, in a hyperlipidemic milieu further activates Tets and increase in 5hmC levels, aggravating *Rac1* transcriptional activation. Mitochondria copy numbers and its DNA transcription are much lower in type 2 diabetes, compared to type 1 diabetes [43]. The retina with high concentration of omega-3 fatty acids, has its own unique fatty acid profile, and is also prone to exacerbated and accelerated damage in a hyperlipidemic environment; Rac1-Nox2-ROS pathway appears to play a significant role in this damage (Figure 2).

This review is focused on Nox2, but the involvement of other members of the Nox family in the development of diabetic retinopathy cannot be ruled out. Genome wide association study and metanalysis have now shown an association between *Nox4* gene and the severity of retinopathy in type 2 diabetic patients [89]. Pharmacological inhibition of Nox4 reduces the severity of experimental retinal vasculopathy [90]. Nox1, Nox4 and Nox5 are implicated in increased vascular permeability and neovascularization [91].

## 6. Therapeutic Implications

NADPH oxidases are the major source of cytosolic ROS, and several leading laboratories have suggested focusing on Nox2 as a therapeutic target to prevent excessive cytosolic ROS generation in many diseases including cancer [92]. In the pathogenesis of diabetic retinopathy, activation of Rac1 is an early event, and sustained activation of Rac1-Nox2-ROS damages the mitochondria, initiating a self-propagating a vicious cycle of free radicals, making it an attractive target for therapeutic intervention. As mentioned above, Nox2 is a multi-component enzyme, which allows multiple avenues to target this enzyme, such as Rac1 and its GEFs and GDI, and interaction between its various subunits and their assembly. Pharmacological approaches to regulate Rac1 activation by focusing on its GEFs has gained attention. Experimental models have shown that the inhibitors of its GEFs- Tiam1 and Vav2, inhibit the development of diabetic retinopathy via regulating Rac1-Nox2 signaling [31,50]. Pharmacological and molecular inhibitors of posttranslational modifications, and the enzymes associated with such modification provide another opportunity to regulate Rac1 activation [57,93]. Apocynin, an inhibitor which impedes binding of p47phox with p22phox, diphenyleneiodonium chloride (DPI), pefabloc, proline-arginine rich antimicrobial peptide and new peptide inhibitors have been developed to particularly target NADPH oxidases, such as gp91 ds-tat and novel nonpeptide VAS2870 [94,95]. Chemicals that inhibit generation of ROS provide considerable benefits over general antioxidants such as vitamin E, which appears to be less efficient due to various properties, including decreased bioavailability. This makes blocking the assembly of NADPH oxidase subunits to reduce the function, and downstream effects of Nox2, as attractive therapeutic targets; various peptide and non-peptide inhibitors are known that mainly operate by disrupting the association of Nox complex assembly [95,96]. The main focus should be to develop an inhibitor with increased efficiency and specificity of binding with the protein subunit, and comprehensive studies are needed on the molecular subunit structures to be targeted and their effects on interactions with other subunits present downstream in the Nox2 complex. For example, Rac1-Nox2 signaling is inhibited by statins, which inhibits 3-hydroxy-3-methylglutaryl coenzyme A, and a population-based cohort study in Taiwan has shown promising results in decreasing the risk retinopathy and its progression [97]. However, additional studies are needed to ascertain the link between statins and diabetic retinopathy. Furthermore, since Nox2 is also stimulated by the renin–angiotensin–aldosterone system, and both experimental and clinical studies have shown that the regulation of the renin–angiotensin system improves retinal neurovascular pathology [67,98], which is observed before vascular pathology [99]. This opens up the use of such inhibitors to regulate Nox2 stimulation and prevent the development and progression of diabetic retinopathy.

As detailed above, a combination of hyperlipidemia and hyperglycemia further activates Rac1-Nox2 signaling, potentiating and exacerbating ROS production, and accelerating mitochondrial damage. Management of hyperlipidemia/obesity in pre-diabetic patients by healthy lifestyle and weight management, without undermining the importance of maintaining their hyperglycemic control, has potential to prevent the development of diabetic retinopathy.

Epigenetic modifications play a major role in its transcriptional activation of *Rac1* in the pathogenesis of diabetic retinopathy, and since epigenetic modifications can also be reversed, targeting such modifications is another possible therapeutic option. Many demethylating agents, e.g., azacytidine and decitabine, are now in clinics to treat myelodysplastic syndromes; however, their non-specific effects make them less desirable [100,101]. Hydralazine and procainamide are also in clinical trials for tumors [102]. As transcription of *Rac1* is also regulated by histone modifications, and histone modifications can regulate DNA methylation and vice versa, targeting histone modification machinery becomes another desirable target, and regulation of Suv39H1-H3K9 trimethylation could prevent dynamic DNA methylation process.

Activation of Rac1-Nox2 is an early event in the pathogenies of diabetic retinopathy, and sustained activation of Rac1-Nox2-ROS damages the mitochondria, initiating a self-propagating a vicious cycle of free radicals. As mentioned above, Nox2 is also associated with other abnormalities seen in the early stages of diabetic retinopathy including increase in inflammatory mediators, leukostasis and blood-retinal barrier damage, and inhibition of its activation will spare the retina from these metabolic abnormalities and prevent further progression of this blinding disease. Rac1-Nox2 activation is also associated with aberrant retinal neovascularization, and its inhibition, during the advanced stages of retinopathy has potential to slow down neovascularization, a hallmark of proliferative diabetic retinopathy.

## 7. Conclusions

As described above, Nox2 plays many contributory roles in the development and progression of diabetic retinopathy. Its activation in early stages of the disease increases cytosolic ROS, damages blood retinal barriers and increases inflammatory mediators. As the duration of hyperglycemia increases, accumulation of cytosolic ROS damages the mitochondria and the damaged mtDNA-electron transport chain initiates a vicious cycle of ROS, which continues to self-propagate. In the later stages of diabetic retinopathy, via regulating angiogenic signaling pathways of VEGF and hypoxia-inducible factors, Nox2 contribute to the neovascularization. This makes targeting Rac1 Nox2 signaling an attractive therapeutic option to prevent the development and progression of diabetic retinopathy. Several inhibitors of Nox2 and Rac1 (and its GEFs) are in experimental or clinical trials for other diseases, and their possible use in diabetic retinopathy will be a welcoming sign for diabetic patients. In addition, since hyperlipidemia further exacerbates and potentiates hyperglycemia-induced Rac1-Nox2 activation, future diabetic retinopathy treatment modalities should also include maintenance of a healthy lipid profile for a diabetic patient, which will help them alleviate the risk of losing their vision.
